# Stem cell factor and NSC87877 combine to enhance c-Kit mediated proliferation of human megakaryoblastic cells

**DOI:** 10.1371/journal.pone.0206364

**Published:** 2018-11-02

**Authors:** Pawan Kumar Raghav, Ajay Kumar Singh, Gurudutta Gangenahalli

**Affiliations:** Division of Stem Cell and Gene Therapy Research, Institute of Nuclear Medicine and Allied Sciences (INMAS), Timarpur, Delhi, India; University of South Alabama Mitchell Cancer Institute, UNITED STATES

## Abstract

Enhancement of hematopoietic stem cells (HSCs) proliferation is a central aim in bone marrow transplantation (BMT). A stem cell factor (SCF) and c-Kit mediated extracellular signaling trigger proliferation of HSCs. This signaling is negatively regulated by protein tyrosine phosphatases (PTPs), SHP-1 and SHP-2. Although NSC87877 (N) is known to inhibit SHP-1/SHP-2, c-Kit-mediated HSCs proliferation by inhibiting SHP-1/SHP-2 has not been reported. This study investigated the combined effect of SCF (S) and N in c-Kit mediated proliferation and underlying mechanisms. The growth of human megakaryoblastic cell line, MO7e and HSCs, upon treatment with S and N alone, and in combination was assessed by PrestoBlue staining. The expression of c-Kit, phosphorylated c-Kit, SHP-1/SHP-2 and HePTP inhibition using S and N treatment were evaluated in the MO7e cells. Megakaryoblast cell proliferation was determined by quantification of Ki-67^+^, S-phase, BrdU^+^ and CFDA-SE^+^ cells using flow cytometry. The combination of S and N leads to enhanced cell growth compared with either S or N alone. Collectively, the results reveal a novel mechanism by which S in combination with N significantly enhances proliferation of human megakaryoblast cells. The pretreatment of N before S enhances proliferation of cells than S alone. This promising combination would likely play an essential role in enhancing the proliferation of cells.

## Introduction

Hematopoietic stem cells (HSCs) recovery after bone marrow transplantation (BMT) has been determined very low and can be overcome by enhancing the proliferation [1]. The proliferation of HSCs prominently begins with the c-Kit pathway [2]. This pathway involves the SCF (S) binding with the extracellular domain of c-Kit leads to receptor dimerization [3]. The cascade of autophosphorylation initiated at intracellular c-Kit tyrosine residues, which also recruits several other binding partners that promote or inhibits cell growth [2,4]. Therefore, S and c-Kit are the two essential partners required in hematopoiesis, and their nonappearance reported fatal [5]. Protein kinase C (PKC) is a family of serine/threonine kinases that are essential regulators of c-Kit [6]. Stimulation of c-Kit with soluble S results in PI3K dependent activation of phospholipase D [7] that released phosphatidic acid and dephosphorylated to produce an activator of PKC, diacylglycerol (DAG). The PKC modulates the tyrosine kinase phosphorylation activity of c-Kit. Down-modulation of c-Kit activity by PKC involves dual mechanisms. Activation of PKC phosphorylates S741 and S746 in the kinase insert region of c-Kit, this leads to inhibition of kinase activity [8]. The suppressors of cytokine signaling-1 (SOCS-1) has been identified as an interactor with c-Kit [9]. Targeted deletion of SOCS-1 leads to a reduced proliferative response via c-Kit upon S stimulation [10].

The SHP-1 and SHP-2 are the protein tyrosine phosphatases (PTPs) that are mostly expressed in the HSCs [11]. SHP-1 diminishes the proliferation signaling by dephosphorylation of the CSF1, EPO, IL-3, and c-Kit receptors either directly or indirectly [12]. Both SHP-1 and SHP-2 negatively modulates c-Kit signaling by interacting with pY570 and pY568 respectively [12]. Although, a chemical molecule, NSC87877 (N) is known to inhibit the enzymatic activity of several PTPs like SHP-1 (IC_50_ = 0.355μM), SHP-2 (IC_50_ = 0.318μM), and hematopoietic protein tyrosine phosphatase (HePTP) (IC_50_ = 7.745 μM) [13]. Besides, several mutations in c-Kit have also been reported which enhances proliferation but are cancerous [14]. However, this abnormal proliferation is not inhibited by SHP-1 or SHP-2 even after associated with mutated (D816V) c-Kit [15]. Importantly, the ability of SHP-2 to associate with activated c-Kit is markedly reduced by the Y568F mutation but remains unaffected by the Y570F mutation. Moreover, expression of c-Kit bearing phenylalanine substitutions at either Y568 or Y570 is associated with enhanced proliferation in response to S.

Several studies have been reported wherein the proliferation through c-Kit detected insignificant due to the low level of c-Kit expression [16]. Efforts have been made to enhance the proliferation by treating cells with recombinant S [17]. This treatment is costly because of using S at high concentration for obtaining significant proliferation. Previously, no study has been reported to evaluate the quantitative proliferation through c-Kit by inhibiting SHP-1 and SHP-2. Therefore, this study investigated the role of S and N (alone and in combination) in mediating proliferation of human megakaryoblastic cells, MO7e which might be used for the expansion of cells. Besides, the expression of c-Kit, phosphorylated c-Kit, PTPs inhibition were also evaluated. All experiments were performed by synchronizing MO7e cells in serum-starved medium (RPMI only) for 20h.

## Materials and methods

### Chemicals and reagents

The purchased chemicals and kits from respective company used were Propidium Iodide (PI) (Calbiochem, #537059), Ribonuclease A (RNase A, Biotech, #9001-99-4.), ethanol (Sigma Aldrich, #1009832500), PBS (Sigma Aldrich, #P4417), fetal bovine serum (FBS) (Gibco, USA), antibiotic (Himedia, #A018), Iscove's Modified Dulbecco's Medium (IMDM) (Sigma Aldrich, #I7633), N (Calbiochem, #565851), Human S (Sigma Aldrich, #57901), BrdU colorimetric cell proliferation kit (Calbiochem, #JA1599), 5(6)-carboxyfluorescein diacetate N succinimidyl ester (CFDA-SE) (Sigma Aldrich, #21888), PrestoBlue (Invitrogen, #A13261), Paraformaldehyde (PFA) (Sigma Aldrich, #P6148), Ki-67 mouse monoclonal IgG antibody (SantaCruz, #23900), Goat Anti-mouse IgG FITC Conjugated (Sigma Aldrich, #F5387), Bovine Serum Albumin (BSA) (AMRESCO, #0332), sodium azide (HiMedia, #GRM1038), saponin (Calbiochem, #558255), StemPro-34 nutrient supplement (Gibco, #10641), CyQuant cell proliferation assay kit (Invitrogen, #35011), Anti-CD117 antibody (c-Kit-PE, Millipore, #10482), Anti-Phosho c-Kit (pY568 and pY570) antibody (Abcam, #ab5616), Rabbit monoclonal Anti-SHP-1/2 (Millipore, #04742), Goat Anti-rabbit IgG-R-PE (Invitrogen, #PZ771MP), Mouse Anti-HePTP (PTPN7) IgG2b_κ_ (Millipore, #04278), Anti-mouse IgG2b FITC (Sigma Aldrich, #SAB3701184).

### Cell culture

Progenitor HSCs isolated from human bone marrow were purchased from Lonza and cultured in IMDM with 2% FBS for 12h before experimenting. Additionally, MO7e cells were obtained as a kind gift from Paulo De Sepulveda, INSERM scientist, Institute National de la Santé et de la Rcherche Médicale, France. The *in vitro* doubling time of MO7e cells is 40h and dependent on the continuous support of growth factors such as GM-CSF, IL-3 and S [16,18–20]⁠. MO7e cells were maintained in RPMI medium with 10% heat-inactivated FBS (complete medium) and 1× antibiotic supplemented with 10ng/mL S at 37°C in 5% CO_2_ humidified atmosphere. Each experiment was performed after MO7e synchronization for 20h using serum-starved (RPMI only without FBS) medium before cells seeding.

### Effect of S and N combination to drive proliferation

1×10^4^ unsynchronized and serum-starved synchronized MO7e cells were resuspended in complete medium in a 96-well transparent plate. These cells were incubated with the S and N in six combinations for 40h, i.e., control (S-N-), N alone (S-N+), S alone (S+N-), both S and N (S+N+), pre-treatment of N 1h before S (Pre), and post-treatment of N 1h after S (Post). Subsequently, PrestoBlue reagent (blue) at 10μL was added to each well and absorbance was measured. Firstly, different concentrations of S (0-80ng/mL), N (0–40μM), and their combinations were used to quantify proliferation in MO7e cells. Later, concentrations of S at 40ng/mL and N at 40μM for MO7e cells were used to assess the proliferation. Also, the proliferation was observed by treating 5×10^3^ HSCs/well with S (40ng/mL) and N (40μM) and incubated for 24h by following the same staining procedure as described earlier.

### Measurement of c-Kit, phospho c-Kit (pY568/570) and PTPs expression

Synchronized MO7e cells (2×10^5^) in each well of 24-well plate containing complete-medium were treated with S and N combinations for 40h. Afterward, cells were harvested and washed with PBS. The cells pellet was incubated in 100μL BSA on ice for 20 minutes to block Fc receptors. CD117 (c-Kit) staining was performed using anti-CD117, a PE-conjugated antibody for 1h at 4°C. The antibody treated cells were washed three times and fixed in 4% PFA at room temperature for 10 minutes followed by three washing with PBS and were analyzed by BD LSRII.

Besides, phosphorylated c-Kit and inhibition of PTPs (SHP-1/2 (both SHP-1 and SHP-2), and HePTP) intracellular staining was performed. The S and N treated MO7e cells were washed with PBS and then fixed in fixative solution (4% PFA in PBS containing 0.1% saponin and 0.5% Tween 20), for 30 minutes at 4°C. Fixed cells were again washed twice with 200μL permeabilization buffer (1% BSA, 0.01% sodium azide in PBS containing 0.5% Tween20 and 0.5% saponin). Then cells were resuspended in 75μL of permeabilization buffer followed by incubation for 30 minutes at 4°C. Primary antibody (0.1μg) of rabbit polyclonal IgG antiphospho c-Kit, rabbit monoclonal anti-SHP-1/2, and mouse anti-HePTP (PTPN7) IgG2b_κ_ antibodies containing 25μL of permeabilization buffer were added in respective tubes and incubated for 30 minutes on the ice. Unbound primary antibodies were washed out using three washes of permeabilization buffer. The secondary antibody at 0.2μg, goat anti-rabbit IgG-R-PE for phospho c-Kit and SHP-1/2 and anti-mouse IgG2b FITC for HePTP was added to the cells containing 100μL permeabilization buffer. Further, cells were incubated for 30 minutes at 4°C in the dark and washed thrice with 200μL permeabilization buffers. Finally, cells were resuspended in staining buffer (1% BSA and 0.01% sodium azide in PBS) and acquired using flow cytometer, LSRII.

### Ki-67 analysis

Synchronized MO7e cells (2×10^5^) were plated in 24-well plate containing 500μL complete-medium and 1× antibiotics. The plated cells were treated with S and N using six combinations, S-N-, S-N+, S+N-, S+N+, Pre, and Post, incubated for 40h. Following this, cells were washed twice with PBS and fixed using fixative solution, for 30 minutes at 4°C. Fixed cells were spun at 500×g for 5 minutes and washed with 200μL permeabilization buffer (1% BSA, 0.01% sodium azide in PBS containing 0.5% Tween20 and 0.5% saponin) twice. Cells were resuspended in 75μL of permeabilization buffer and incubated for 30 minutes at 4°C. Primary antibody (0.1μg) for Ki-67 antigen was added separately in respective tubes with 25μL permeabilization buffer and incubated for 30 minutes on ice. Unbound primary antibodies were washed out by washing with permeabilization buffer thrice. The secondary antibody (0.2μg) was added to the cell suspension in 100μL permeabilization buffer. Subsequently, cells were incubated for another 30 minutes at 4°C in the dark and washed three times with 200μL permeabilization buffers. Successively, cells were resuspended in 400μL of PI staining solution and acquired by the flow cytometer, LSRII. Auto (without staining), primary antibody staining only and secondary antibody staining samples were used as controls to check the non-specific binding. The analysis was subjected to high-resolution cell-cycle analysis using the Flowing software.

### Cell cycle analysis to identify the cycling state of the MO7e cells

The cell cycle status of MO7e cells treated with S and N evaluated the distribution of cells in different cell cycle phases. The cell cycle analysis of synchronized MO7e cells, (2×10^5^ cells/well) seeded in 24-well plate, incubated for 40h was performed to measure the mitotic index drive through G_1_, S-phase and G_2_ phase. The cell cycle progression, G_1_ to S-phase was observed using S at 40ng/mL and N at 40μM treatment, alone and in combination. Then the cells were washed twice with 1.5 mL PBS (without Mg^2+^ and Ca^2+^). After that, fixed the cells in 1mL PFA and stored at 4°C for 2h. Just before staining, PFA was removed by spinning at 300×g for 10 minutes and washed cells twice in 2mL PBS. Lastly, the pellet was resuspended in 0.5mL PI solution (10μg/mL propidium iodide in 1.1% sodium citrate buffer with 1mg/mL RNase A added) to stain DNA. This treatment was followed by an incubation at 37°C for 20 minutes in the dark before flow cytometry acquisition.

### BrdU colorimetric assay

BrdU chasing was performed to measure the proliferation of MO7e cell treated with S-N-, S-N+, S+N-, S+N+, Pre and Post in 96-well plate for 40h. Synchronized MO7e cells in triplicates were assayed to avoid variation in biological responses at the cellular level. Seeded 100μL of MO7e cells at a density of 2×10^5^ cells/mL into a 96-well plate. Two types of controls were considered, blank (only tissue culture medium) and background (cells without BrdU). This BrdU chasing was performed using BrdU colorimetric cell proliferation kit (Calbiochem) according to the manufacturer's protocol.

### Quantification of total cellular DNA content using CyQuant

CyQuant cell proliferation assay assesses proliferation by quantifying total cellular DNA content [21–23]. Synchronized MO7e 10,000 cells/well were seeded in a 96 well white plate and incubated with S at 40ng/mL and N at 40μM respectively in complete medium for 24h. The cells were stained and measured the fluorescence according to the manufacturer’s protocol.

### CFDA-SE labeling

The 2μL of 5mM CFDA-SE stock was diluted by adding to 1mL of PBS. Afterward, synchronized MO7e cells (4.5×10^6^) were harvested and washed three times with PBS. The 3.6×10^6^ cells were added to the equal volume of CFDA-SE at a concentration of 10μM and incubated at 37°C for 5 minutes at room temperature. Subsequently, cells were washed by diluting labeled cells in 10 volumes of 20°C PBS containing 5% heat-inactivated FBS. The cells were centrifuged at 280×g for 5 minutes at 20°C, discarded the supernatant and washed twice. The CFDA-SE labeled cells were washed two times with PBS and seeded 2×10^5^ cells/mL in RPMI containing 10% FBS. Furthermore, cells were treated with S-N-, S-N+, S+N-, S+N+, Pre, and Post and incubated for 40h.

### Statistical analyses

Results expressed as mean ± SD. Independent two-sided Student's t-test was used to compare two groups. Data were considered statistically significant at a value of *p* <0.05, *p* <0.01, *p* <0.005 and *p* <0.001.

## Results

### S and N combination increases cells proliferation

**Fig 1:**
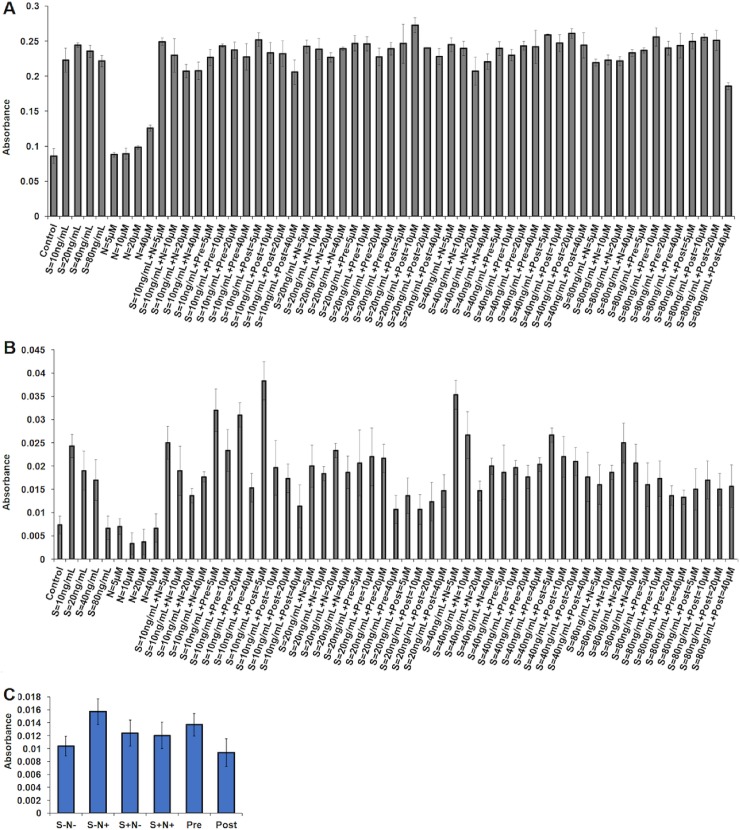
Proliferation assessed by absorbance of PrestoBlue in S and N treated **(A)** unsynchronized MO7e cells, **(B)** synchronized MO7e cells, and **(C)** HSCs at S, 40ng/mL and N, 40μM.

PrestoBlue cell viability reagent evaluated the proliferation of unsynchronized (Fig 1A) and serum-starved synchronized (Fig 1B) MO7e cells treated with different concentrations of S, N and their combination for a duration of 40h. The unsynchronized MO7e cells were remained metabolically active and continued to proliferate irrespective of treatments. However, S+N+, Pre, and Post showed a higher proliferation activity as compared to S alone and N alone. Despite, an increased proliferation was observed for S alone but did not show significant compared to S+N+, Pre and Post measured using PrestoBlue dye reduction. Nonetheless, synchronized cells treated with S at 40ng/mL and N at 40μM revealed obvious differences in their proliferative activities for 40h compared to S alone at 40ng/mL (Fig 1B). As a result, this concentration was used to measure the effect of S at moderate concentration, 40ng/mL, and N at 40μM which also exhibited increased proliferation for N alone treatment among unsynchronized and synchronized proliferation assay. Additionally, a corresponding increased proliferation was observed for S = 40ng/mL+N = 40μM and S = 40ng/mL+Pre = 40μM whereas, high difference was noted between S = 40ng/mL+N = 5μM and S = 40ng/mL+Pre = 5μM (displayed higher proliferation). Besides, elevated proliferation was observed in bone marrow-derived HSCs treated with S at 40ng/mL and N at 40μM (Fig 1C)

### S and N decreases c-Kit but increases phospho c-Kit (pY568/570) expression

**Fig 2:**
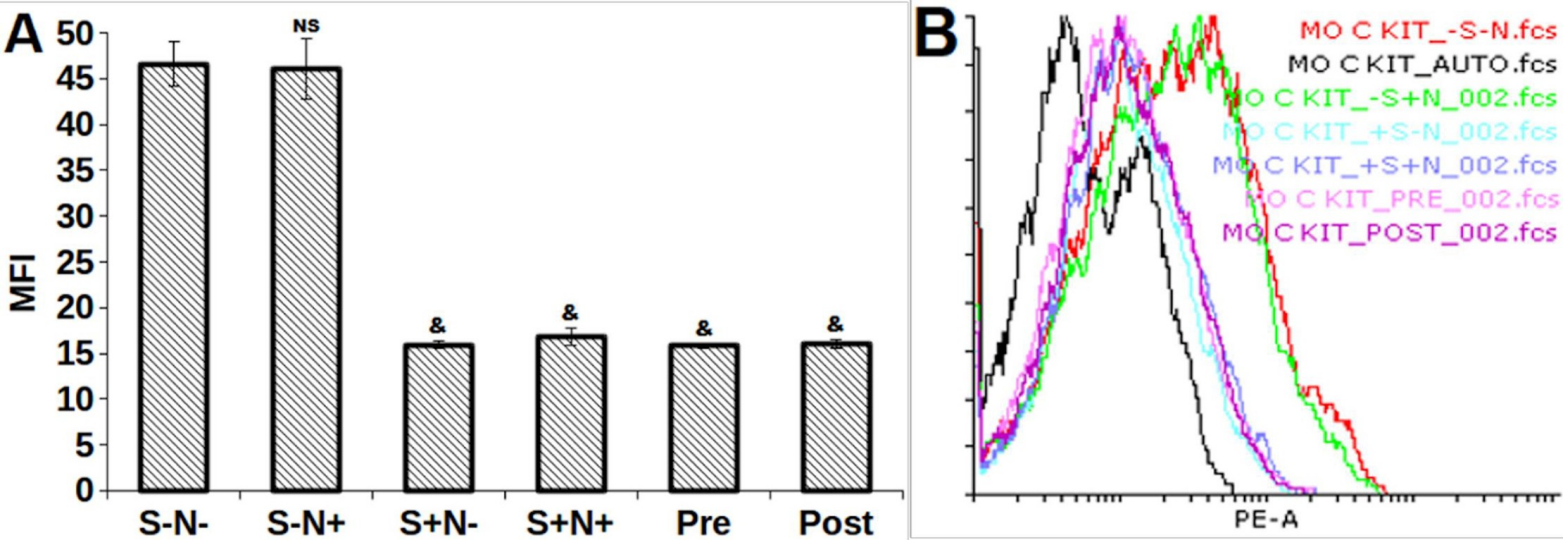
**(A)** Comparative bar graph and, **(B)** overlay histogram of c-Kit expression MFI. *, *p*<0.05; &, *p*<0.001; NS, not statistically significant.

The c-Kit expression on serum-starved MO7e cells was evaluated by flow cytometry analysis (Fig 2A and 2B). A significant decrease was observed in the c-Kit expression on MO7e cells treated with S+N-, S+N+, Pre, and Post as compared to control, confirming that S decreases the c-Kit expression whereas, S-N+ treatment on c-Kit expression remains unaffected.

**Fig 3:**
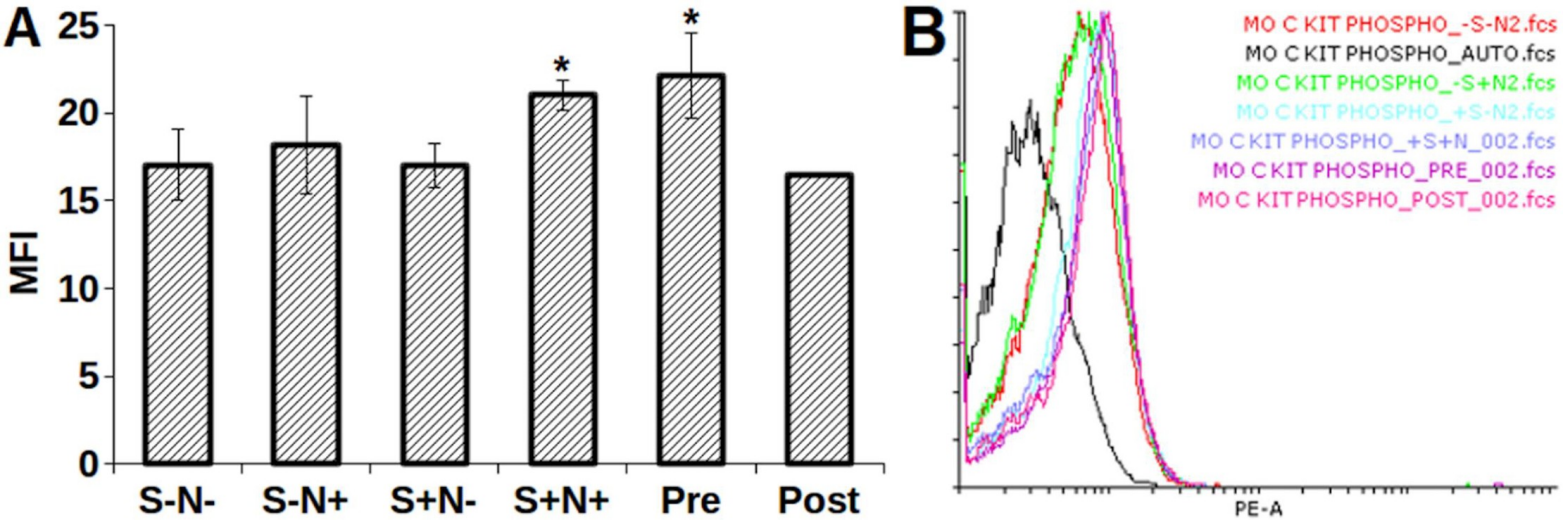
**(A, B)** MFI of c-Kit phosphorylation expression determined on S and N treated MO7e cells at 40h by flow cytometry. *, *p*<0.05; &, *p*<0.001; NS, not statistically significant.

The highest c-Kit phosphorylation status at Y568 and Y570 were detected using antiphospho (pY568/570) antibody in Pre (*p* = 0.024) treated MO7e cells (Fig 3A and 3B) as compared to control (S-N-). Similarly, S+N+ treatment indicated a significantly (*p* = 0.016) effective inhibition of c-Kit dephosphorylating PTPs, SHP-1 and SHP-2.

### S and N combination decreases PTPs expression

**Fig 4:**
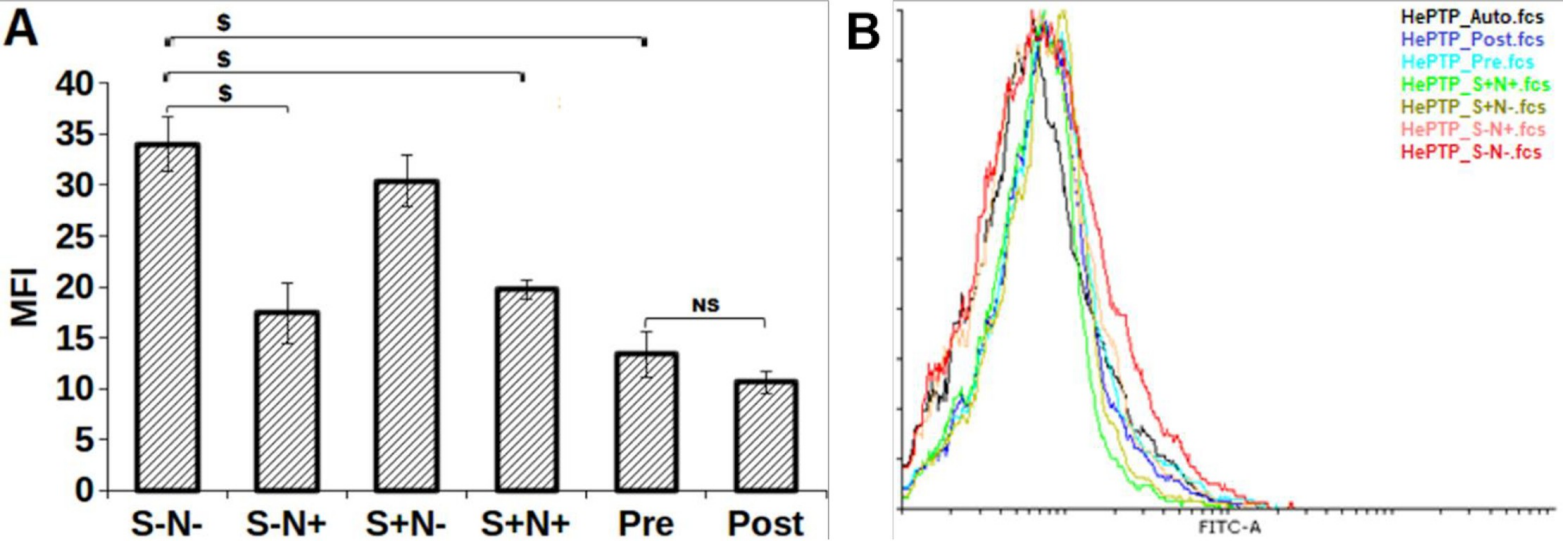
MFI of HePTP inhibition using S and N alone, and in combination **(A)** bar graph, **(B)** overlay histogram. $, *p*<0.005; NS, not statistically significant; *, *p*<0.05.

**Fig 5:**
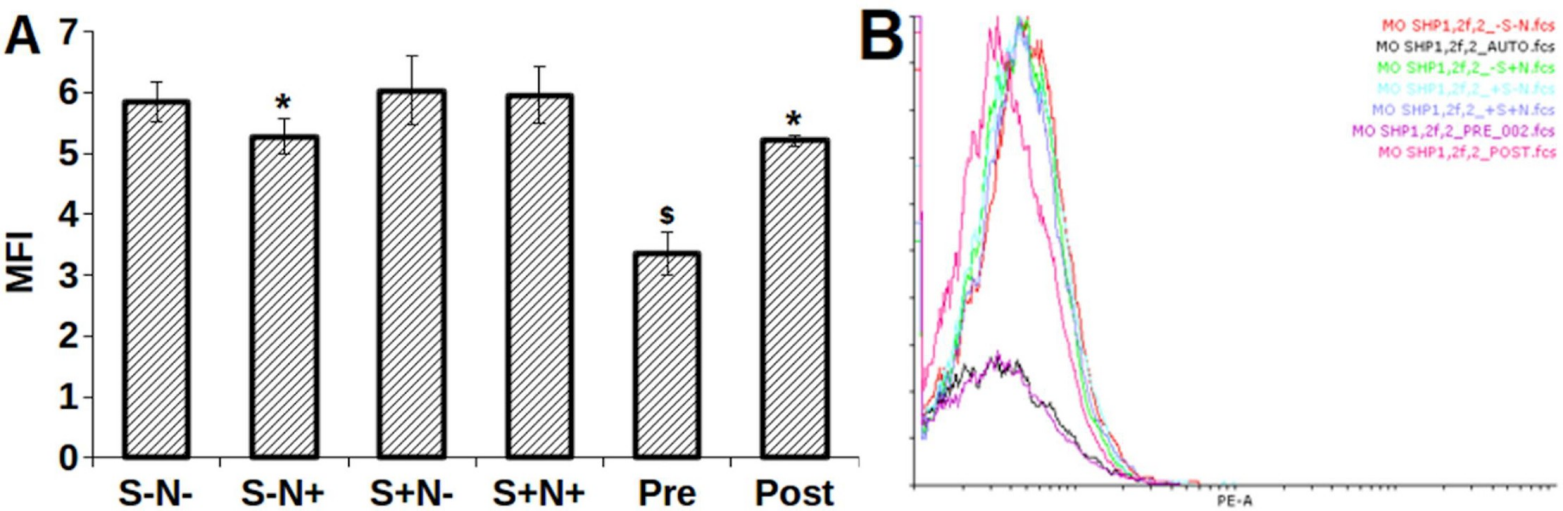
MFI of SHP-1/SHP-2 inhibition by S and N treatment. **(A)** bar graph, **(B)** overlay histogram. $, *p*<0.005; NS, not statistically significant; *, *p*<0.05.

The inhibition of SHP-1, SHP-2, and HePTP expression was identified in S and N treated synchronized MO7e cells. A significant decrease in HePTP expression was observed in S-N+ (*p* = 0.003), S+N+ (*p* = 0.003), Pre (*p* = 0.001) and Post (0.0005) treated cells as compared to S-N- (Fig 4A and 4B). Highest inhibition was observed for Post but found insignificant (*p* = 0.22) compared to S-N- treated cells. Moreover, treatment of Pre (*p* = 00.0043) on MO7e cells exhibited significant inhibition of SHP-1 and SHP-2 using SHP-1/2 antibody (detected both SHP-1 and SHP-2) as compared to S-N- treated cells (Fig 5A and 5B). Likewise, S-N+ (*p* = 0.04) and Post (*p* = 0.015) demonstrated the effective SHP-1/2 inhibition.

### Combination of S and N enhances cell cycle progression

**Fig 6:**
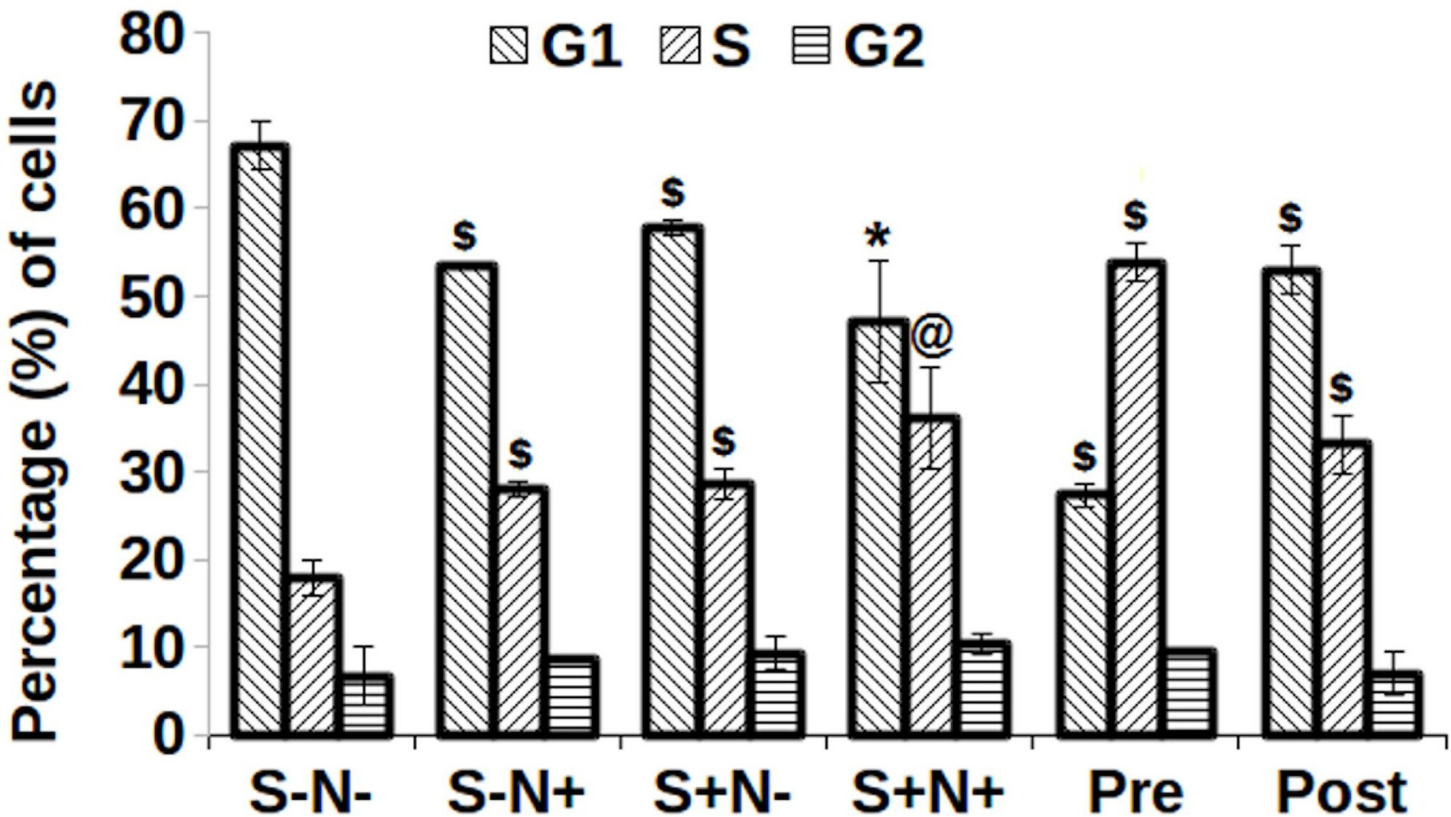
Cell cycle phases of cells derived by treatment of S and N and their combination. $, *p*<0.005; @, *p*<0.01; *, *p*<0.05 shows with respect to their respective control, S-N-.

The cell cycle status and cycling kinetics of S and N treated MO7e cells from prolonged G_0_/G_1_ to S and G_2_ phase were investigated. Strikingly, cells treated with Pre, displayed 53.88% of cells in S-phase, compared to 17.89% by S-N- cells. All treatments exhibited a statistically significant (*p*<0.005) increase in the percentage of cells in S-phase excluding S+N+ (*p*<0.01). In agreement with this observation, the reduction in the percentage of cells in G_1_ phase (Fig 6) was also found to be significant (*p*<0.005), except S+N+ (*p*<0.05) as compared to S-N- treated cells.

### BrdU colorimetric assay assessed enhanced proliferation

**Fig 7:**
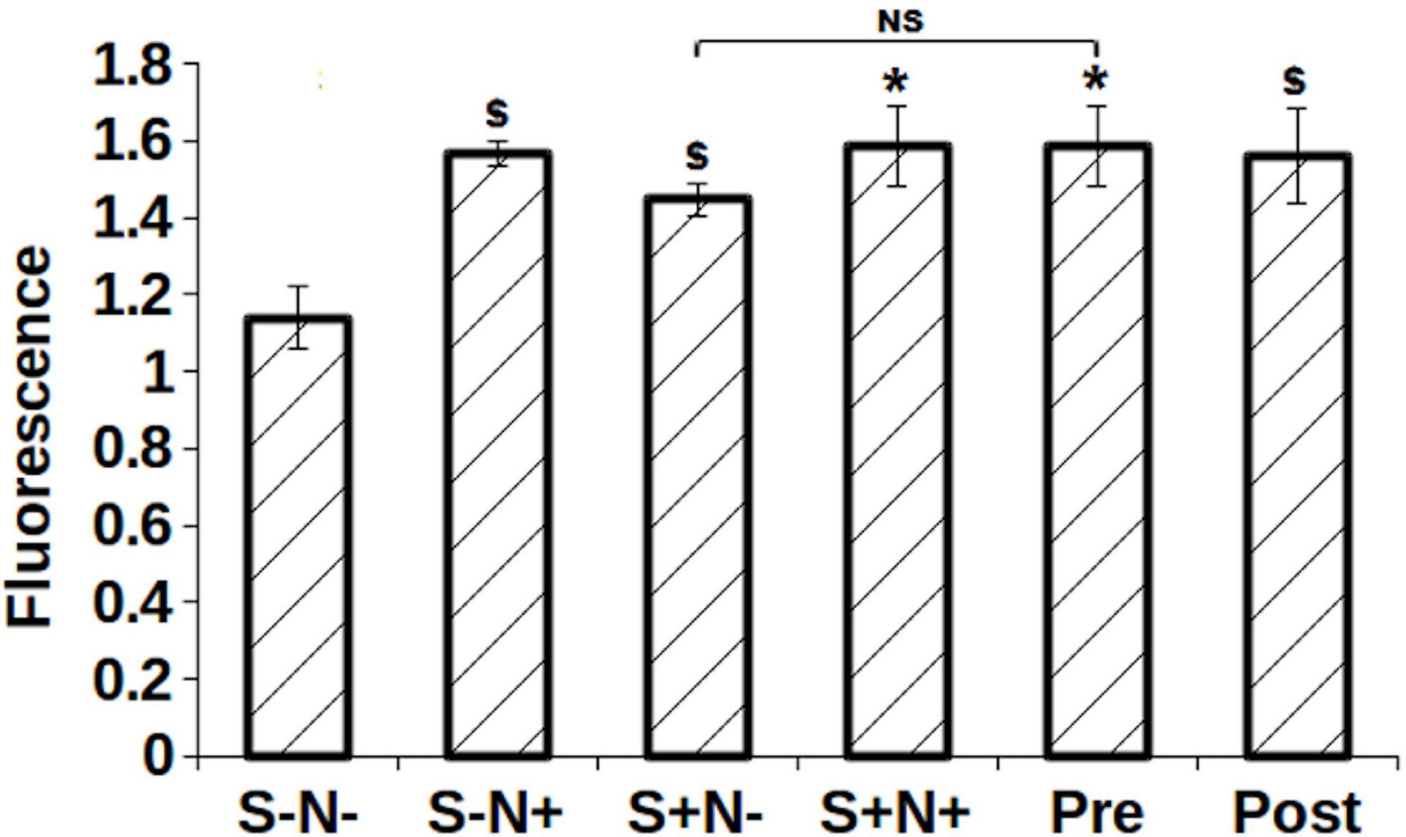
Shows chasing of fluorescently BrdU labeled MO7e cells in S-phase up to 40h. $, *p*<0.005; *, *p*<0.05.

BrdU chasing determined the proliferation by labeled all S-phase MO7e cells during cell cycle progression for 40h upon S and N treatment. A significantly increased proliferation in S-N+ (*p* = 0.0005), S+N- (*p* = 0.002), S+N+ (*p* = 0.01), Pre (*p* = 0.012) and Post (*p* = 0.004) treated cells was observed compared to S-N-, control (Fig 7). However, no significant increase in BrdU labeled S-phase was seen in Pre (*p* = 0.16) treated cells as compared to S+N- treatment.

### Effect of S and N treatment on DNA content

**Fig 8:**
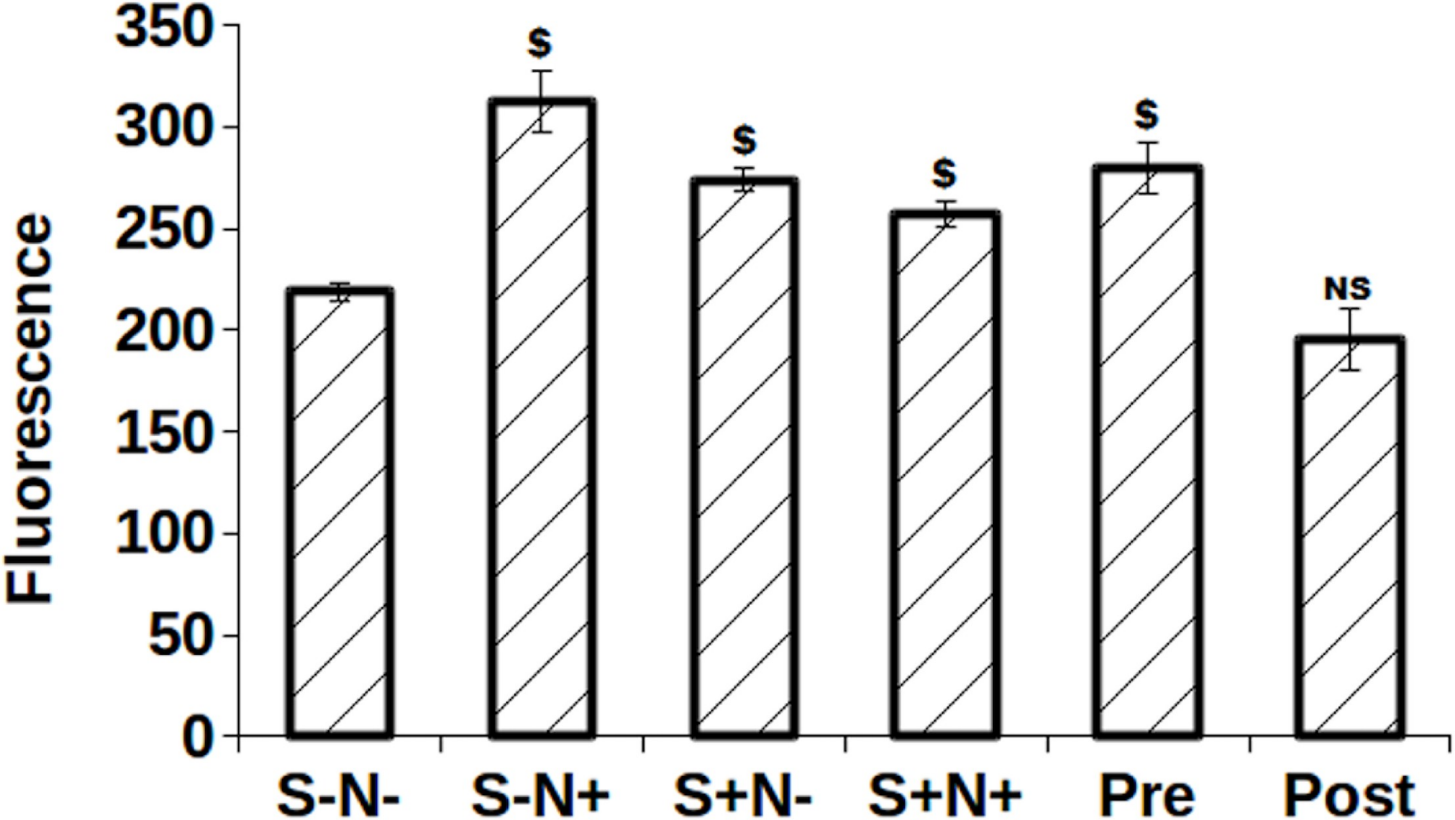
Fluorescence of total DNA content measured by CyQuant $, *p*<0.005; NS, not statistically significant.

The significant difference was detected for S-N+ (*p* = 0.0002), S+N- (*p* = 0.0001), S+N+ (*p* = 0.0005), Pre (*p* = 0.0007) as compared with S-N- treated cells (Fig 8). S-N+ exhibited highest proliferation among all treatments.

### CFDA-SE labeling of MO7e cells shows enhanced proliferation

**Fig 9:**
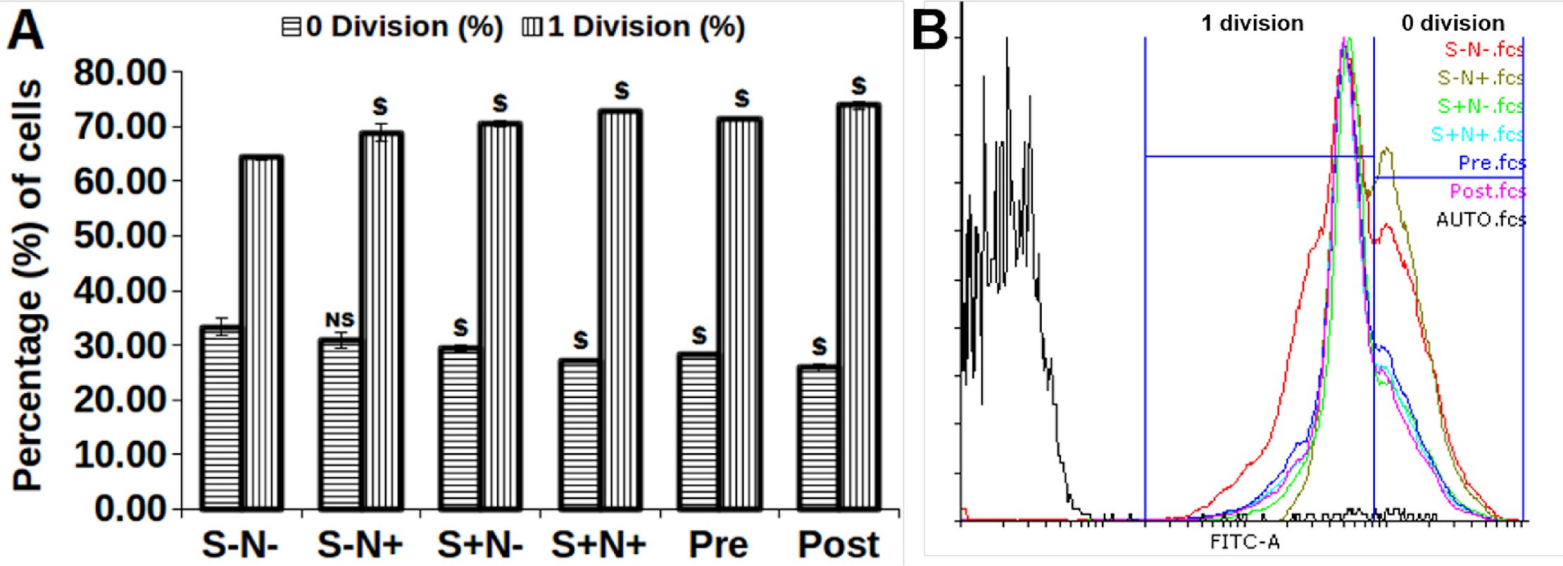
**(A)** A significant increase in cell division, 1 division by S and N treatment for 40h compared to control and decrease in 0 division, **(B)** represents the gated fluorescence of cell’s population in 0 division and 1 division. $, *p*<0.005; NS, not statistically significant.

MO7e cells stained with CFDA-SE evaluated the *in vitro* cell proliferation and to track the number of cell divisions undergone in response to the treatment of S-N-, S-N+, S+N-, S+N+, Pre, and Post (Fig 9A). The cells were found to undergo two divisions, 0 division showed cells with higher CFDA-SE stain (undivided), whereas 1 division displays the first cell division (Fig 9B). The cells treated with S alone, N alone and their combination significantly increased (*p*<0.005) the average number of cells in 1 division after 40h as compared to S-N- treatment. Furthermore, CFDA-SE profiles for 0 division displayed significant decrease (*p*<0.005) in parent CFDA-SE labeled cells (0 division) for S+N-, S+N+, Pre, and Post, while S-N+ demonstrated no significant cell division as compared to S-N- treated cells.

### Enhanced Ki-67 expression confirmed proliferation in MO7e cells

**Fig 10:**
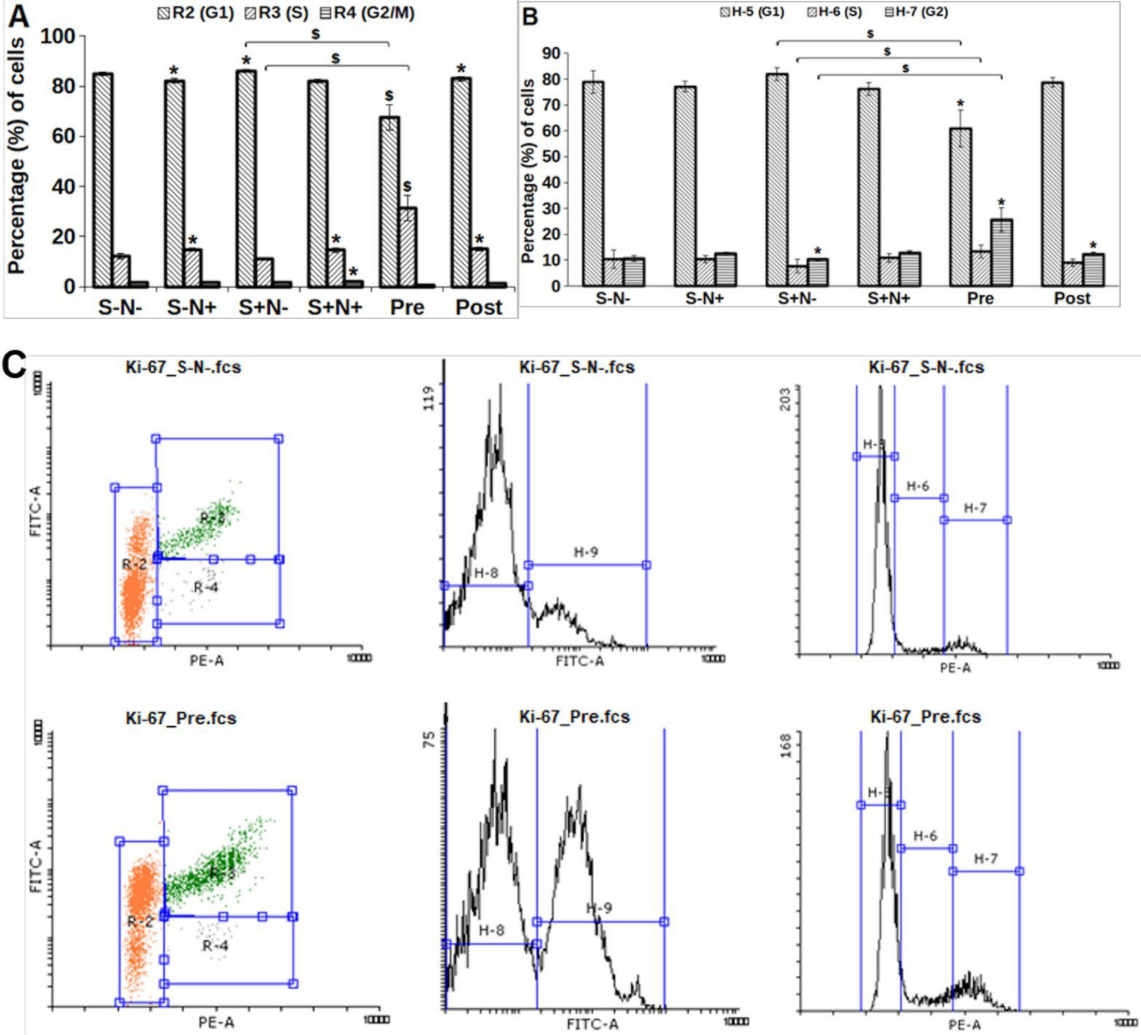
**(A)** Percentage of cells represent the Ki-67 staining, **(B)** percentage of cells in cell cycle phases stained using PI, and **(C)** depicts the gating by applying compensation to separate the Ki-67 (FITC-Area) and PI (PE-Area) population. $, *p*<0.005; NS, *, *p*<0.05.

A significant (*p*<0.005) increased proliferation was determined by observing the expression of the nuclear protein, Ki-67 antigen (Fig 10A), and cell cycle (Fig 10B) for 40h on MO7e cells. A gate was applied to separate the G_1_, S and G_2_/M, and Ki-67 positive population (Fig 10C). The proliferation of treated cells with Pre was significantly (*p* = 0.00031) obtained highest (31.4%) in S-phase as compared to S-N- (12.07%), whereas a significantly (*p* = 0.002) decreased percentage of cells (67.4%) were found in G_1_ phase as compared to 84% for S-N-. Additionally, the Ki-67 expression (*p* = 0.0012) was observed to be upregulated for Pre in S-phase compared to S+N- (11.3%). Besides, cell cycle progression showed increase in 25.64% (*p* = 0.037) of cells in G_2_ phase, decrease of 60.96% (*p* = 0.01) cells in G_1_ phase for Pre, as compared to S-N-, 10.7% (G_2_) and 78.9% (G_1_). The statistical significant percentage of cell was noted for Pre in G_1_ (*p* = 0.004), S (*p* = 0.027) and G_2_ (*p* = 0.002).

## Discussion

**Fig 11:**
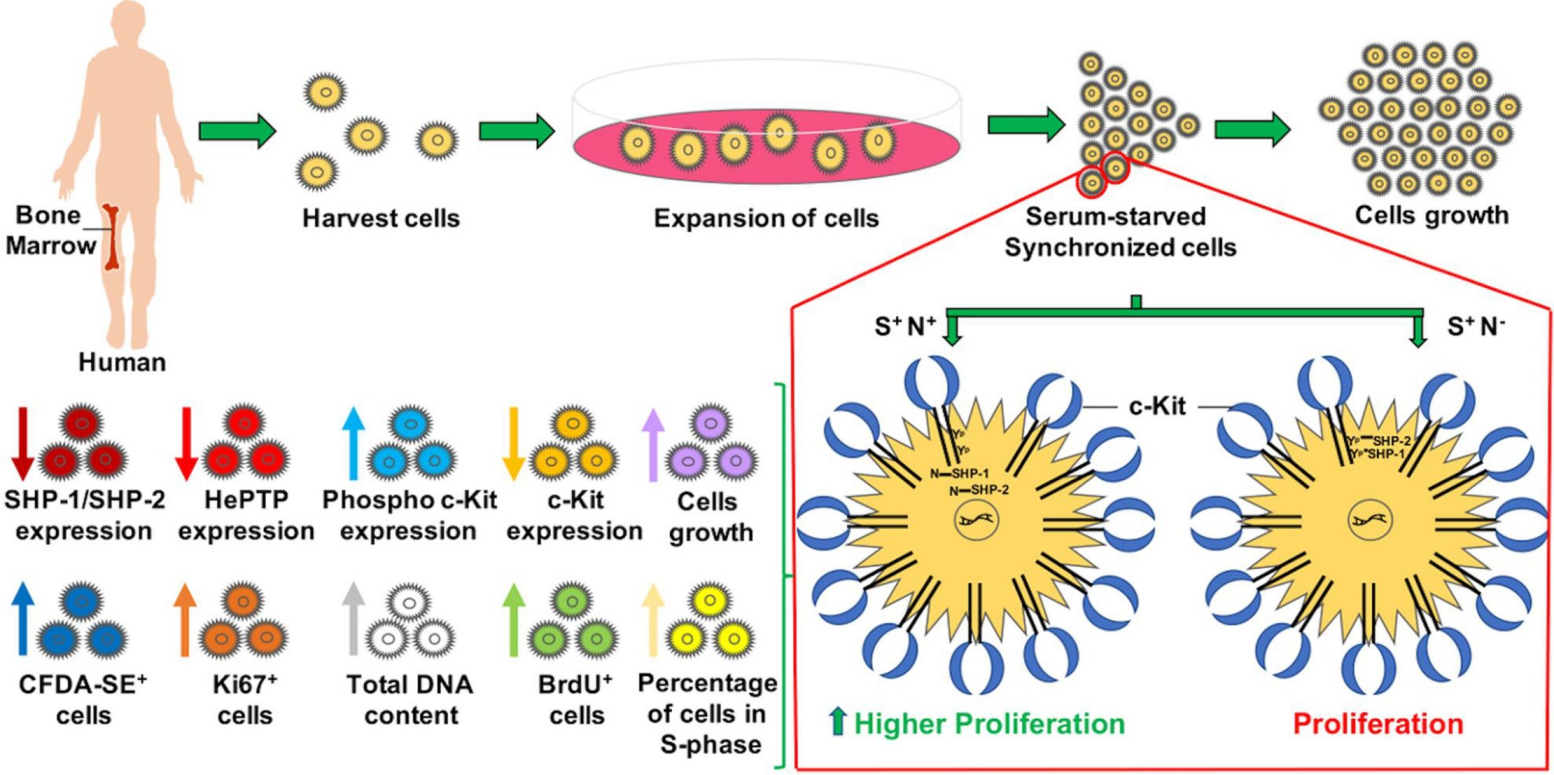
A representation of isolation of bone marrow cells, their expansion and assessment of growth of serum-starved synchronized cells. The synchronized cells treated with SCF (S^+^) only and without the addition of NSC87877 (N^-^) leads to low proliferation whereas, S^+^ N^+^ treatment decreases the c-Kit expression, enhances the c-Kit phosphorylation, and proliferation, evaluated by cell cycle analysis, BrdU, CyQuant, and Ki-67 analysis.

Both c-Kit and SHP-1/SHP-2 mRNA expression was reported in MO7e cells [24–27]. Therefore, to identify the effect of S and N combination on proliferation, an S dependent cell line, MO7e cells were used. MO7e cells proliferate under the influence of growth factors S, GM-CSF, and IL3. This combination generates a promising model to study the S dependent proliferation activated through c-Kit [28]. The schematic representation of work performed is shown in Fig 11. Serum starvation of MO7e cells for 20h before a cell-based assay was performed to synchronize all cells to the same cell cycle phase. The synchronization removed the impact of FBS on the cell cycle of the cells that would respond only to the treatments. Thus, comparative analysis of the proliferation of unsynchronized and synchronized MO7e cells suggested that synchronization is essential to assess the proliferation and further to obtain significant changes. The experiment performed on unsynchronized and synchronized cells were independent to each other. Although, no distinctive proliferation was noted in unsynchronized cells. The highest proliferation was obtained using N at 40μM and S at 20ng/mL for unsynchronized cells.

On the contrary, among S alone, 80ng/mL of S shows the lowest proliferation, but S at 40ng/mL shows moderate concentration between S at 20ng/mL and 80ng/mL. Furthermore, the proliferation of synchronized cells achieved using N at 5μM and 40μM seems very close to each other. So, a higher concentration of N at 40μM was chosen which is also concurred with N at 40μM for unsynchronized cells. Moreover, S at 10ng/mL shows the highest proliferation and lowest at 80ng/mL amongst S treatment. Thus, a higher concentration of S, 40ng/mL was preferred, instead, S = 10ng+Post = 5μM which exhibits the highest proliferation. The impact of S and N treatment was also determined in HSCs and was found to increase the number of HSCs for S-N+ treatment. These results correlated with the proliferation assays performed with synchronized MO7e cells. It has been reported that S at concentrations of 10-100ng/mL significantly decreases the expression of c-Kit [29,30]. The effect of S and N treatment on c-Kit expression confirmed that c-Kit expression substantially decreases on S treatment, while no change was identified for S-N+ treatment.

Furthermore, an increased c-Kit phosphorylation was identified in S+N+, and Pre treated MO7e cells. The increased autophosphorylation of c-Kit was recognized by effective inhibition of SHP-1/SHP-2 and HePTP. Thus, SHP-1/2 and HePTP inhibition was evaluated and indicated that treatment of Pre leads to c-Kit phosphorylation. This result suggested that SHP-1/2 inhibition has not be facilitated in S+N- treatment, thus showed lower c-Kit phosphorylation which in turn leads to diminutive proliferation. The cell cycle result shows the higher percentage of cells in S-phase for cells treated with Pre which revealed faster cell cycle progression. These findings suggested that previous S and N combination might play an unrecognized role in proliferation. BrdU result is consistent and correlated with cell cycle progression and PrestoBlue based proliferation results. The BrdU finding was further confirmed by measuring fluorescence of DNA content using CyQuant. The CyQuant result indicates that the addition of S might have caused a delay in cell cycle progression from S-phase. Besides, CFDA-SE estimated the proliferation by calculating the division of labeled cells and showed that all treated cells significantly increased first division as compared to control. This result, unequivocally demonstrated that both S and N treated cells undergo self-renewing showed by *in vitro* divisions. The Ki-67 and BrdU results confirmed that Pre treatment enhances the proliferation as compared to other treatments.

## Conclusion

In summary, the proliferation was assessed in a human megakaryoblastic cell line, MO7e which possessed high c-Kit expression. The present study also investigated the augmentation of proliferation by assessing c-Kit expression, c-Kit phosphorylation, and PTPs inhibition using S and N alone, and in combination in the MO7e cells. Moreover, the combined effect of Pre-treatment of N before S (Pre) enhances proliferation as compared to S alone treated synchronized cells that have high expression of c-Kit. This study would likely be used to enhance the proliferation of megakaryocytes cells to increase the number of platelets for patients having low platelet count or to enhance HSCs cells growth.

## Abbreviations

HSCs: Hematopoietic Stem Cells
PTPs: Protein Tyrosine Phosphatases
SHP-1: Src Homology 2 Domain Containing Phosphatase-1
SHP-2: Src Homology 2 Domain Containing Phosphatase-2
HePTP: Hematopoietic Tyrosine Phosphatase
PTKs: Protein Tyrosine Kinases
Stem cell Factor: SCF (S)
NSC87877: N
Control: S-N-
N alone: S-N+
S alone: S+N-
Both S and N: S+N+
Pre-treatment of N 1h before S: Pre
Post-treatment of N 1h after S: Post
