# Correction: Stem cell factor and NSC87877 combine to enhance c-Kit mediated proliferation of human megakaryoblastic cells

**DOI:** 10.1371/journal.pone.0210133

**Published:** 2018-12-28

**Authors:** Pawan Kumar Raghav, Ajay Kumar Singh, Gurudutta Gangenahalli

There are errors in the Author Contributions. Pawan Kumar Raghav was incorrectly listed as having contributed to Conceptualization; Gurudutta Gangenahalli should be listed as contributing to Conceptualization.

The complete, correct Author Contributions are: Conceptualization: GG. Data curation: PKR. Formal analysis: PKR. Funding acquisition: GG. Investigation: GG. Methodology: PKR. Project administration: GG. Resources: AKS. Supervision: GG. Validation: PKR. Visualization: PKR. Writing–original draft: PKR. Writing–review & editing: PKR, AKS, GG.
